# Characteristics and complications of fracture in older adults with chronic kidney disease: a cross-sectional study

**DOI:** 10.1186/s13018-022-03253-8

**Published:** 2022-08-06

**Authors:** Yao Meng, Mingming Fu, Junfei Guo, Zhiqian Wang, Yingze Zhang, Zhiyong Hou

**Affiliations:** 1 Department of Geriatric Orthopedics, The Third Hospital of Hebei Medical University, Shijiazhuang, 050051 Hebei People’s Republic of China; 2 Department of Orthopaedic Surgery, The Third Hospital of Hebei Medical University, Shijiazhuang, 050051 Hebei People’s Republic of China; 3 NHC Key Laboratory of Intelligent Orthopaedic Equipment (The Third Hospital of Hebei Medical University), Shijiazhuang, Hebei 050051 People’s Republic of China; 4 Chinese Academy of Engineering, Beijing, 100088 People’s Republic of China

**Keywords:** Chronic kidney disease, Fracture, Older adults, Cardiovascular calcification, Hemoglobin

## Abstract

**Background:**

The aim of this study was to analyze the clinical characteristics of older fracture patients with chronic kidney disease (CKD) and to determine the risk factors of perioperative cardiovascular complications.

**Methods:**

We retrospectively reviewed clinical data of older fracture patients with CKD admitted to the Third Hospital of Hebei Medical University from January 2016 to October 2021. The data we collected included baseline characteristics and complications. We finally determined the risk factors of perioperative cardiovascular complications by using logistic regression.

**Results:**

We ended up enrolling 224 patients, and there were 91 (40.6%) males and 133 (59.4%) females, with a median age of 79 years. 80–84 years old was the age group with high incidence of fracture. The majority of fracture occurred indoors (130 cases, 58.0%) and morning (98 cases, 43.8%). Hip fracture was most common (183 cases, 81.7%), of which femoral neck fracture (101 cases, 45.0%) was the most prevalent. The most common comorbid condition was hypertension (171 cases, 76.3%), and anemia was the most common complication (148 cases, 66.1%). Age ≥ 80 years (OR = 2.023, 95% CI 1.110–3.688), previously combined with cardiovascular calcification (OR = 1.901, 95% CI 1.047–3.451) and admission hemoglobin level < 100 g/L (OR = 3.191, 95% CI 1.744–5.838) were independent risk factors of perioperative cardiovascular disease (CVD).

**Conclusion:**

It was especially necessary to enhance fracture prevention for CKD. Patients whose age older than 80, hemoglobin less than 100 g/L on admission and have previous cardiovascular calcification are more likely to develop perioperative CVD. Such patients require reasonable decisions during the perioperative period to avoid the occurrence of CVD.

## Background

As the population ages, the number of the older over 60 years of age will reach 2 billion by 2050 [1]. Fracture causes serious threats to elderly’s health and life quality. CKD is increasingly common in elderly. With the worsen of kidney function, the fracture rates of the older fallers also increased [2]. Renal osteodystrophy (ROD) has become an important public health problem in the aging societies.

The risk of death after fracture is related to perioperative cardiovascular events [3, 4]. Calcification of blood vessels and valves will cause high risk of CVD among chronic kidney disease patients [5–7]. Therefore, cardiovascular calcification will greatly increase the risk of dying from cardiovascular events during the perioperative period among fracture patients with CKD [8, 9].

At present, there are many studies on end-stage renal disease with fracture, but the research on CKD combined with fracture is rare. Therefore, we researched the people with CKD and analyzed their clinical characteristics and the risk factors of CVD.

## Methods

### Patients and groups

This was a retrospective study of 224 adults over 65 years of age with fracture and previous CKD who were hospitalized in the Third Hospital of Hebei Medical University from January 2016 to October 2021. This study was declaration of Helsinki compliant, and the Institutional Review Committee of the Third Hospital of Hebei Medical University supported us in doing this research. Since this is a retrospective study, we gave up the informed consent of the respondents and protected the confidentiality of them by anonymizing their data.

The collected data of interest included demographic data such as gender and age, the characteristics of fracture and the complications in the perioperative period. From 65 to 100 years, patients were divided into seven age groups at intervals of 5 years. The time of fracture occurrence was recorded as dawn (0:00–6:00), morning (6:00–12:00), afternoon (12:00–18:00) and night (18:00–24:00). The place where the fracture occurred included indoor and outdoor. The fracture types were divided into hip (femoral neck, intertrochanter) and non-hip fracture.

### Inclusion and exclusion criteria

Inclusion criteria were as follows: (1) 65 years of age or older; (2) with a previous diagnosis of CKD; (3) with a definite diagnosis of fracture by imaging; (4) with complete data.

Exclusion criteria were as follows: (1) high energy trauma fracture (car accident, fall from height, etc.); (2) old fracture; (3) long-term administration of glucocorticoids or selective estrogen receptor modulators; (4) have a history of diseases that affect bone metabolism, such as tuberculosis, malignant tumors, and Cushing’s syndrome.

There were two orthopedic physicians and one internal medicine physician forming an investigation team. Two orthopedic doctors performed the image reading. The agreement between the two orthopedic doctors was 95%. The internal medicine physician conducted quality control and regularly conducted sampling inspections on the reading results.

### Disease definition

The diagnosis of cardiovascular calcification was based on chest radiography to detect aortic and coronary calcification, and cardiac ultrasound to detect calcification of heart valves. If the patient has calcification in at least one examination site, the patient was assessed as having cardiovascular calcification: mitral valve, aortic valve, aorta, or coronary artery [10, 11].

### Statistical analysis

Data were analyzed by IBM SPSS Statistics version 25.0, and the statistically significant level was set at *P* < 0.05. All data are based on Kolmogorov–Smirnoff to test for normality. We used the mean ± standard deviation to represent the data that conformed to the normal distribution, and non-normally distributed variables were represented by the median and interquartile range. Two sample Welch t test or nonparametric test was used for continuous variables, and Chi-square or nonparametric test for categorical variables. Categorical variables were expressed in frequency and percentage. The data with a *P* < 0.10 were analyzed with logistic regression.

## Results

### Baseline characteristics

From January 2016 to October 2021, 224 fracture patients with CKD who satisfy the inclusion criteria were enrolled in the study. These patients included 133 (59.4%) women and 91 (40.6%) men, with a median age of 79 years. The most common age group was 80–84 years old, with a total of 51 cases (22.8%). A total of 171 patients were presenting with hypertension, accounting for 76.3%, other comorbidities included cardiovascular calcification (47.8%), chronic heart failure (46.9%), diabetes (42.0%) and old cerebral infarction (38.8%). Of the 224 patients, 49 (21.9%) from dialysis patients, and 175 (78.1%) from patients received no dialysis. CKD stage 5 (78, 34.8%) and CKD stage 3 (78, 34.8%) patients accounted for the highest proportion (Table 1).

**Table 1:** Baseline characteristics of elderly patients with CKD and fracture

Variables	Cases	Percentage
Gender, *n* (%)		
Male	91	40.6
Female	133	59.4
Age, years	79 (14)	
Age groups, *n* (%)		
65–69	38	17.0
70–74	39	17.4
75–79	37	16.5
80–84	51	22.8
85–89	37	16.5
90–94	19	8.5
95–100	3	1.3
Comorbidity, *n* (%)		
Hypertension	171	76.3
Cardiovascular calcification	107	47.8
Chronic heart failure	105	46.9
Diabetes	94	42.0
Old cerebral infarction	87	38.8
History of dialysis, *n* (%)		
Dialysis	49	21.9
Non-dialysis	175	78.1
CKD stage, *n* (%)		
CKD 1	5	2.2
CKD 2	15	6.7
CKD 3	78	34.8
CKD 4	48	21.4
CKD 5	78	34.8
Fracture location, *n* (%)		
Hip fracture	183	81.7
Non-hip fracture	41	18.3

*n*(*) represents the median (interquartile range)

**Fig. 1:**
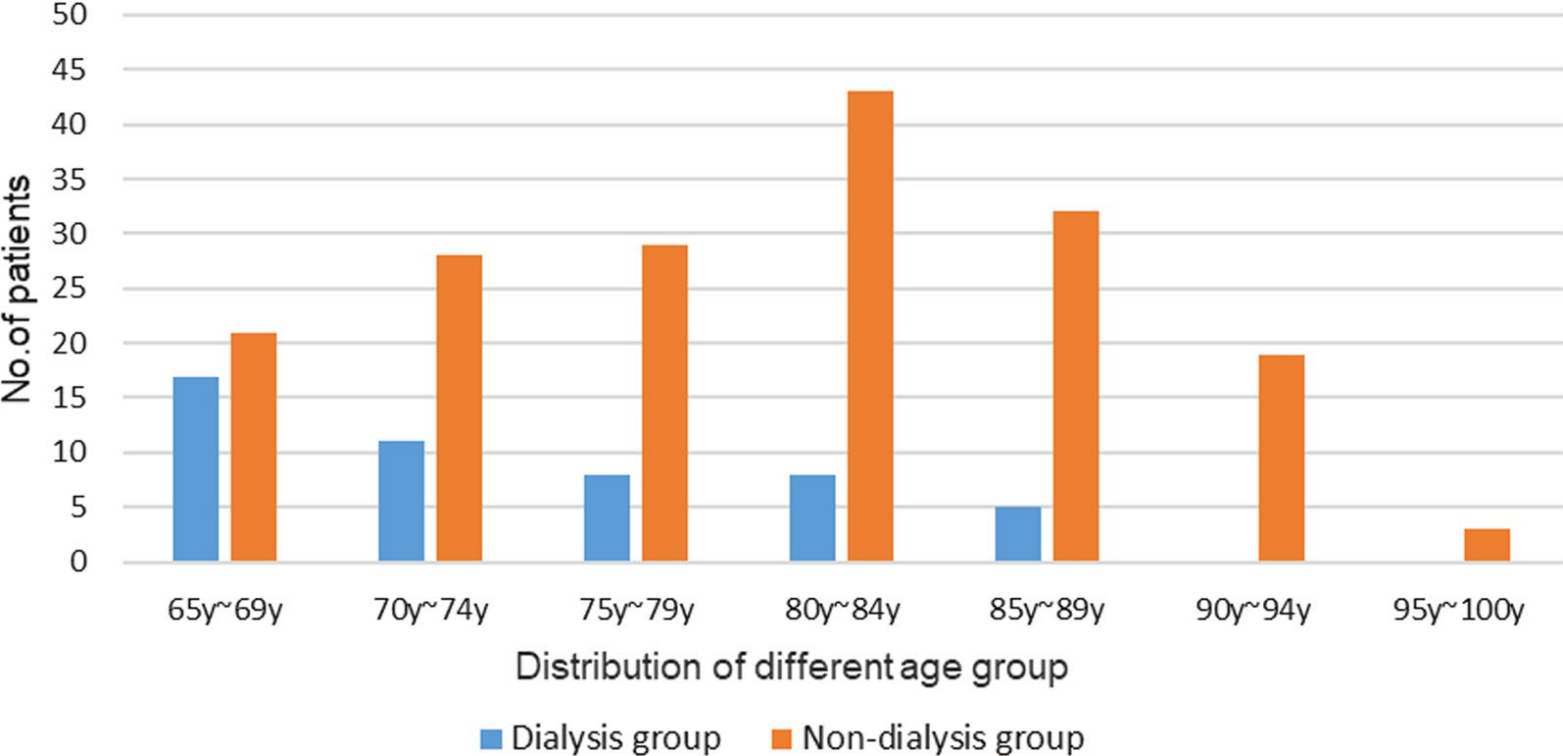
Composition of age and history of dialysis in elderly patients with CKD and fracture

The age groups distribution of dialysis and non-dialysis patients was different statistically (*P* < 0.05). The most prevalent age group of dialysis patients was 65–69 years, and 80–84 years was the predominant age group of non-dialysis patients. The specific distribution of characteristics can be seen in Fig. 1.

### Place and time of fracture occurrence, and types of hip fracture

Among the data when and where fracture occurred, most patients developed nine fracture indoors (130 cases, 58.0%) and in the morning (98 cases, 43.8%). There were 101 (45.0%) femoral neck fracture and 82 (36.6%) femoral intertrochanteric fracture (Table 2).

**Table 2:** The distribution of place, time of fracture occurrence and type of hip fracture

Variables	Cases (*n*)	Percentage (%)
Place		
Indoor	130	58.0
Outdoor	94	42.0
Time		
Dawn (00:00–6:00)	20	8.9
Morning (6:00–12:00)	98	43.8
Afternoon (12:00–18:00)	77	34.4
Evening (18:00–24:00)	29	12.9
Type of hip fracture		
Femur neck fracture	101	45.0
Femur intertrochanteric fracture	82	36.6

### Perioperative complications

Patients with anemia in the perioperative period accounted for the highest proportion (148 cases,66.1%) .147 cases (65.6%) with cardiovascular complications, of which acute heart failure was the most frequent (122 cases, 54.5%), followed by arrhythmia (62 cases, 27.7%) and myocardial infarction (19 cases, 8.5%). Other complications included hypoalbuminemia (112 cases, 50.0%), deep vein thrombosis of the lower extremities (78 cases, 34.8%), electrolyte disorder (72 cases, 32.1%), serous cavity effusion (45 cases, 20.1%) and pulmonary infection (41 cases, 18.3%) (Table 3).

**Table 3:** Perioperative complications of patients with CKD and fracture

Variables	Cases (*n*)	Percentage (%)
Cardiovascular complications	147	65.6
Myocardial infarction	19	8.5
Acute heart failure	122	54.5
Arrhythmia	62	27.7
Anemia	148	66.1
Deep vein thrombosis of the lower extremities	78	34.8
Electrolyte disorder	72	32.1
Hypoalbuminemia	112	50.0
Pulmonary infection	41	18.3
Serous cavity effusion	45	20.1

### Analysis of risk factors of CVD

The differences between the patients with and without CVD during the perioperative period are shown in Table 4. Factors including age, types of fracture, combined with cardiovascular calcification, and hemoglobin on admission were *P* < 0.1. The logistic regression showed that age ≥ 80 years (OR = 2.023, CI = 1.110–3.688, *p* = 0.021), previous combined with cardiovascular calcification (OR = 1.901, CI = 1.047–3.451, *p* = 0.035) and hemoglobin on admission < 100 g/L (OR = 3.191, CI = 1.744–5.838, *p* = 0.000) were important independent risk factors of perioperative CVD in fracture patients with CKD (Table 5).

**Table 4:** The association of CVD with gender, age, fracture location, comorbidities, and number of hemoglobin on admission

Variables	Total	CVD		*χ*^2^	*P* value
		Yes (*n* = 147)	No (*n* = 77)		
Gender				0.006	0.936
Male	91	60	31		
Female	133	87	46		
Age (years)				7.038	0.008
< 80	124	72	52		
≥ 80	100	75	25		
Fracture location				4.617	0.032
Hip fracture	183	126	57		
Non-hip fracture	41	21	20		
Combined with Hypertension				0.005	0.942
Yes	171	112	59		
No	53	35	18		
Combined with Cardiovascular calcification				3.647	0.056
Yes	107	77	30		
No	117	70	47		
Hemoglobin on admission				14.370	0.000
< 100 g/L	109	85	24		
≥ 100 g/L	115	62	53

**Table 5:** Multivariate analysis of preoperative variables associated with CVD (multinomial logistic regression; (Enter)

CVD	B	Wald	Sig	Exp (*B*)	95% confidence interval for Exp (*B*)	
					Lower limit	Upper limit
Age ≥ 80	0.705	5.296	0.021	2.023	1.110	3.688
Cardiovascular calcification	0.642	4.456	0.035	1.901	1.047	3.451
Hemoglobin < 100 g/L	1.160	14.179	0.000	3.191	1.744	5.838

## Discussion

In our study of 224 older adults, we examined characteristics and complications of fracture of patients with CKD. We have found that elderly CKD with fracture was more common in women, 80–84 years old, and people with hypertension. Dialysis patients combined with fracture were more common in younger age groups. Patients with CKD stage 5 had the highest rates of fracture. The type of fracture was mainly hip fracture. Most patients had fracture indoors and in the morning. Anemia accounted for the highest proportion of perioperative complications. Risk factors of CVD in the perioperative period consisted of age ≥ 80 years old, previous combined cardiovascular calcification, and hemoglobin < 100 g/L on admission.

We concluded that the number of women suffered from fracture was more than that of men, and the result was the same as the conclusion of some research works [12]. In addition to the reduced estrogen during menopause, the female CKD patients more commonly have high parathyroid hormone (PTH), leading to loss of cortical bone [13]. There is also evidence that women are more susceptible to CKD [14] and women are an independent risk factor for fracture in hemodialysis patients [15]. Therefore, in CKD patients with fracture, the result that there are more women than men is no exception [16]. Among the patients, we included 80–84 years old CKD with fracture accounted for the largest proportion. As early as the results of a large population study in the USA from 1985 to 2005, it was concluded that the common age of renal failure with hip fracture was 75–84 years. This result overlapped with our results [17]. Most studies believed that advancing age was the driver for the increase in incidence rates of fracture [18, 19]. However, in our study, dialysis patients combined with fracture were more common in younger age groups compared with non-dialysis patients, which might be related to the worse kidney function of dialysis patients, the decline in the function of various organs of the body and the deterioration of life activity ability. As a result, younger dialysis patients were prone to low-energy falls and hip fracture. For such patients, regardless of age, we cannot relax our vigilance.

Across all comorbidities, hypertension accounted for the highest proportion. This was due to the fact that hypertension is one of the most frequent underlying disorders of the elderly. The loss of urinary calcium leads to a decrease in bone quality, and when blood pressure fluctuates, it is easy to cause dizziness and increase the risk of falls [4]. It has also been suggested that patients with hypertension will experience a decrease in calcium bone absorption when treated with calcium channel blockers [20] and a risk factor for falls from orthostatic hypotension when treated with Angiotensin-converting enzyme inhibitor (ACEI) [21]. Our findings indicated that chronic heart failure was the second most common comorbidity, with a total of 105 cases. The conclusion had been verified in Gere Sunder-Plassmann’s research [22, 23]. Growing evidence shows that diabetes mellitus will increase the risk of fracture after a fall, and it may be related to the transient amaurosis or dizziness [24–27]. In our study, patients with CKD stage 5 had the highest rates of fracture (78, 34.8%). The risk of hip fracture increased with severity of kidney diseases [2, 7, 28, 29]. Some researchers have also confirmed that the prevalence of renal failure combined with fracture is as high as 98% in dialysis patients and 84% in CKD3-5 stages [5]. Therefore, patients with severe chronic renal insufficiency are more likely to have ROD.

In our study, elderly people with CKD were more likely to develop fracture indoors and in the morning. One plausible explanation is that dialysis treatment maintains renal function, but also causes physical weakness by the loss of amino acids and other nutrients during dialysis, finally causes skeletal muscle weakness, dizziness, fatigue or slow gait speed and restrict patients’ range of activities indoors in order to avoid outdoor unsafe factors [30, 31]. Another reason that can be explained is CKD patient usually suffers from restless legs syndrome. The calf often feels uncomfortable at night, which causes the patient to overactive at dawn or in the morning to relieve leg pain, and ultimately increase the risk of falling. Therefore, for the older adults with CKD, they should have as many escorts around them as possible, using crutches, guardrails and other measures to prevent falls and keep the floor in the home and the dialysis room dry and tidy. We also showed that hip fracture was the most common type, and most studies have yielded same results [32]. One of the possible explanations of this is people with a higher risk of fracture often have poor neuromuscular function [33, 34]. Moreover, the cross-sectional area of the vastus lateralis muscles will be substantially reduced in the elderly, thus increasing the incidence of hip fracture [35]. In addition, the hip as a long-term weight-bearing site makes the bone more vulnerable to damage, and the hip is usually used as the stress point when most patients fall.

Anemia occupied the highest proportion of perioperative complications in our study, which is related to the reduction in erythropoietin and myelosuppression caused by the long-term accumulation of metabolic waste. In addition, hip fracture as the most common fracture location leads to massive blood loss [36]. Although, in our study, anemia was studied as a perioperative complication, analysis of our data confirmed patients who had hemoglobin on admission < 100 g/L had increased the incidence of CVD. Therefore, long-term chronic anemia can be used to explain other complications, especially the cardiovascular system. Seydou Sy et al. pointed out in the latest study in 2021 that anemia was closely related to myocardial hypertrophy [37]. As CKD patients are often complicated with sodium and water retention in the body, chronic anemia and hypoxia, the heart has a long-term compensatory function to ensure sufficient oxygen supply to the peripheral circulation. Overload of the heart and myocardial hypertrophy will ultimately increase the risks of heart failure and myocardial infarction in the perioperative period. They accounted for 54.5% and 8.5% in our data, respectively. Conversely, heart failure leads to decreased renal perfusion and exacerbates renal failure. Anemia, an important factor in aggravating heart and renal failure, will lead to tissue hypoxia, peripheral vasodilation, sympathetic nervous system and RAAS system excitation and constrict renal blood vessels, aggravate myocardial oxygen consumption. Heart failure, renal failure and anemia activate each other, creating a vicious cycle of damage [38]. So some scholars have proposed that chronic anemia is one of several risk factors of cardiac complications [22] and the concept of cardiorenal anemia syndrome [38, 39].

CKD leads to congestive heart failure by increasing sodium and water retention in the body. Anemia will lead to tissue hypoxia, peripheral vasodilation, sympathetic nervous system and RAAS system excitation and constrict renal blood vessels, aggravate myocardial oxygen consumption, and become an important factor in aggravating heart and renal failure. Heart failure, renal failure and anemia activates each other, creating a vicious cycle of damage.

Many investigators have confirmed that cardiovascular event is the main cause of death in patients with CKD [40, 41]. When the kidney function stage is lower than CKD stage 2, the risk of cardiovascular events will increase as the stages of renal failure increase [42–44]. Hypertension as the most common comorbidities causes the decrease in left ventricular function by increasing cardiac afterload and myocardial oxygen consumption, which ultimately makes patients suffer from high incidence of cardiovascular complications. Therefore, for patients with CKD after traumatic stress, surgeons not only need to solve the problem of fracture, but also deal with the underlying diseases that can cause perioperative complications, such as correcting anemia on admission and controlling hypertension [44]. CKD is a leading contributor to electrolyte disturbance, and electrolyte disturbance is the leading underlying cause of arrhythmia. High extracellular potassium causes prolongation of the action potential duration, which makes the heart more prone to bradyarrhythmias, such as sinus arrest, sinus bradycardia, and ventricular fibrillation [45]. Therefore, correcting serum electrolyte disturbance from the decline of renal function is still the key to reducing perioperative complications in patients with fracture.

Cardiovascular calcification is common among healthy elderly. All arteries can be calcified especially small arteries in hemodialysis patients [45, 46]. This is because patients with CKD are usually accompanied by abnormalities in blood calcium, blood phosphorus, PTH and other bone metabolism indicators [47]. In patients with early renal insufficiency, the fibroblast growth factor-23 (FGF-23) produced by bone cells can be increased, which can inhibit the production of PTH, reduce the absorption of calcium and phosphorus in the intestine, and pass Klotho synergistic action to promote the effect of urinary phosphorus excretion [48]. Blood phosphorus in patients with early renal failure is usually in the normal range, but as renal function deteriorates, the lack of kidney Klotho will reduce the effect of FGF-23 in inhibiting PTH, on the one hand, and eventually lead to excessive release of PTH, which accelerates bone resorption, and intracellular Ca overload ultimately accelerates cardiovascular calcification [48]. On the other hand, as the kidney’s ability to excrete phosphorus decreases, blood phosphorus will counteract the effect of FGF-23 and gradually become higher than normal [49, 50] and induce vascular calcification by activating pro-inflammatory molecules in vascular smooth muscle cells [47]. In addition, FGF23 and Klotho themself are also related to vascular calcification. Klotho has a protective effect against calcification, and its defects in CKD will accelerate cardiovascular calcification [51]. FGF23 is an inhibitor of vascular calcification [52]. In addition, some researchers found decreased bone mass and extensive vascular calcification in mice knocking out FGF23 and Klotho genes. This fact also strengthened the view that there is a common pathway between bone and vascular metabolism [53]. Even the patients with earlier stages of CKD and with normal renal function, vascular calcification can lead to cardiovascular events, shorten life expectancy, and increase mortality [54]. Therefore, for older patients with CKD, surgeons routinely perform chest and abdomen X-rays and echocardiography after the patient being admitted to the hospital, which can effectively detect the degree of calcification of blood vessels and valves, and avoid perioperative CVD.

The fracture elderly with CKD is still a large group. Our data showed that it is important for clinicians to make reasonable decisions in the perioperative management of fracture patients with CKD and avoid the occurrence of perioperative CVD.

### Limitations

First of all, the collection of retrospectively designed data has certain limitations. For example, it is not completely accurate for us to use clinical information collected by one hospital to infer the overall clinical characteristics. Secondly, we collected patients who did not choose dialysis because of subjective factors. In addition, we lack some meaningful indicators, such as bone density, parathyroid hormone and vitamin D collection. At the same time, we have no statistics on the perioperative medications and surgical treatment methods of all the participants.

## Conclusion

Our research results emphasize the need to enhance fracture prevention for CKD patients. Particular attention should be paid to avoiding CVD during the perioperative period for patients whose age older than 80, hemoglobin less than 100 g/L on admission and have previous cardiovascular calcification.

## Data Availability

The data used to support the findings of this study are available from Zhiqian Wang upon request.
